# Editor’s introduction to this issue (G&I 18:3, 2020)

**DOI:** 10.5808/GI.2020.18.3.e25

**Published:** 2020-09-25

**Authors:** Taesung Park

**Affiliations:** Department of Statistics, Seoul National University, Seoul 08826, Korea

In this issue, there are eight articles: six Original Articles, one Application Note, and one article in the category of Genome Archives. The first original article, by the group of Kim et al. (Soongsil University, Seoul, Korea) proposed a novel method for the analysis of single-cell RNA sequencing. Specifically, their article described a semi-automatic method that calculates a normalized score for each cell type based on a user-supplied cell-type–specific marker gene list. Second, Liju et al. (Madras Diabetes Research Foundation, Chennai, India) identified associations of genetic variants with early-onset of type 2 diabetes in a South Indian population. Although the sample size was not large, the association of the *HHEX* variant rs1111875 was successfully demonstrated for the first time in a South Indian population.

The third article, by Ferdous et al. (Mawlana Bhashani Science and Technology University, Tangail, Bangladesh), provided a molecular characterization and functional annotation of a hypothesized protein of *Streptomyces coelicolor* A3(2), which is a Gram-positive soil bacterium known for the production of several antibiotics used in various biotechnological applications. The application of several bioinformatics tools successfully revealed the characteristics of this hypothesized protein from the genome of *S. coelicolor*, including its structure, function, and homologous proteins. The fourth article, by the group of Sa et al. (Kangwon National University, Chuncheon, Korea), presented a comparative gene expression analysis regarding seed pigmentation in maize by comparing differently expressed genes from three inbred lines, including a pigment-accumulating seed type (CM22) and non-pigmented seeds (CM5 and CM19).

Recent active research efforts have produced many articles on the coronavirus disease 2019 (COVID-19) pandemic. In this issue, there are two articles related to COVID-19. The first one, by Sohpal (Beant College of Engineering & Technology, Gurdaspur, India) presents a computational analysis of the genome of severe acute respiratory syndrome coronavirus 2 (SARS-CoV-2, the virus that causes COVID-19) with the genome of the Middle East respiratory syndrome-related coronavirus. Using molecular evolutionary genetic analysis (MEGA) from the National Center for Biotechnology Information (NCBI) for statistical analysis, the best substitution pattern and transition/transversions (R) were compared. The final Research Article was by Apio et al. (Seoul National University, Seoul, Korea), which presents the 95% confidence intervals for the SARS-CoV-2 antibody retention rate for the Korean. The most conservative 95% confidence interval estimation showed that as of 00:00 on September 15, 2020, there were at least 32,602 undetected cases of COVID-19 in Korea.

The one article in the Genome Archives categories, by Islam et al. (Sher-e-Bangla Agricultural University, Dhaka, Bangladesh) presented the sequencing and annotation of the complete mitochondrial genome (16,597 bp in size) of a threatened labeonine fish, *Cirrhinus reba*, collected from the Khulna region of Bangladesh.

The Application Note, by Park and his students (Ewha Womans University, Seoul, Korea) described the initiation of the first *Genomics & Informatics* Annotation Hackathon (GIAH) event, focusing on improving earlier versions of the full-text corpus of *Genomics & Informatics* by semi-automatically detecting and correcting PDF-to-text conversion errors and optical character recognition errors.

